# Supplementary material for: Respondents with more extreme views show moderation of opinions in multi-year surveys in the USA and the Netherlands
Source: Commun Psychol. 2023 Dec 2;1:37. doi: 10.1038/s44271-023-00034-9 (PMC11331986; doi:10.1038/s44271-023-00034-9)
Supplement: Supplementary file 2 — Supplementary Information [file 44271_2023_34_MOESM2_ESM.pdf]

Supplementary Information

Supplementary Table 1

Attitude items, Study 1: General Social Survey (GSS)

Attitude	Items	Response options	Internal consistency
Gender egalitarianism	A working mother can establish just as warm and secure a relationship with her children as a mother who does not work.	1=strongly agree, 2=agree, 3=disagree, 4=strongly disagree *(reverse-coded)*	Wave 1: α=.65<br>Wave 2: α=.63<br>Wave 3: α=.64
	A preschool child is likely to suffer if his or her mother works.	1=strongly agree, 2=agree, 3=disagree, 4=strongly disagree	
	It is much better for everyone involved if the man is the achiever outside the home and the woman takes care of the home and family.	1=strongly agree, 2=agree, 3=disagree, 4=strongly disagree	
	Family life often suffers because men concentrate too much on their work.	1=strongly agree, 2=agree, 3=neither agree nor disagree, 4=disagree, 5=strongly disagree	
Support for affirmative action	Some people say that because of past discrimination, blacks should be given preference in hiring and promotion. Others say that such preference in hiring and promotion of blacks is wrong because it discriminates against whites. What about your opinion -- are you for or against preferential hiring and promotion of blacks?	1=strongly favors, 2=not strongly favors, 3=not strongly opposes, 4=strongly opposes *(reverse-coded)*	Wave 1: *r*=.36<br>Wave 2: *r*=.36<br>Wave 3: *r*=.40
	Irish, Italians, Jewish and many other minorities overcame prejudice and worked their way up. Blacks should do the same without special favors.	1=strongly agree, 2=agree, 3=neither agree nor disagree, 4=disagree, 5=strongly disagree	
Support for redistribution	Some people think that the government in Washington ought to reduce the income differences between the rich and the poor, perhaps by raising the taxes of wealthy families or by giving income assistance to the poor. Others think that the government should not	1=government should… 7=government should not. *(reverse-coded)*	-

	concern itself with reducing this income difference between the rich and the poor. Here is a card with a scale from 1 to 7. Think of a score of 1 as meaning that the government ought to reduce the income differences between rich and poor, and a score of 7 meaning that the government should not concern itself with reducing income differences. What score between 1 and 7 comes closest to the way you feel?		
Support for contraception for teens	Do you strongly agree, agree, disagree, or strongly disagree that methods of birth control should be available to teenagers between the ages of 14 and 16 if their parents do not approve?	1=strongly agree, 2=agree, 3=disagree, 4=strongly disagree *(reverse-coded)*	-
Support of gays/lesbians	What about sexual relations between two adults of the same sex--do you think it is always wrong, almost always wrong, wrong only sometimes, or not wrong at all?	1=always wrong, 2=almost always wrong, 3=wrong only sometimes, 4=not wrong at all	Wave 1: *r*=.69<br>Wave 2: *r*=.70<br>Wave 3: *r*=.70
	Homosexual couples should have the right to marry one another.	1=strongly agree, 2=agree, 3=neither agree nor disagree, 4=disagree, 5=strongly disagree *(reverse-coded)*	
Disapproval of children corporal punishment	Do you strongly agree, agree, disagree, or strongly disagree that it is sometimes necessary to discipline a child with a good, hard spanking?	1=strongly agree, 2=agree, 3=disagree, 4=strongly disagree *(reverse-coded)*	-
Permissive sex attitudes	Do you think it is wrong or not wrong if a man and a woman have sexual relations before marriage?	1=always wrong, 2=almost always wrong, 3=wrong only sometimes, 4=not wrong at all	Wave 1: *r*=.37<br>Wave 2: *r*=.37<br>Wave 3: *r*=.37
	What if they are in their early teens, say 14 to 16 years old? In that case, do you think sex relations before marriage are always wrong, almost always wrong, wrong only sometimes, or not wrong at all?	1=always wrong, 2=almost always wrong, 3=wrong only sometimes, 4=not wrong at all	
Justification of sexual infidelity	What is your opinion about a married person having sexual relations with someone other than the marriage partner--is it always wrong, almost	1=always wrong, 2=almost always wrong, 3=wrong only	-

	always wrong, wrong only sometimes, or not wrong at all?	sometimes, 4=not wrong at all

Note. Items were reverse-coded when necessary. For multiple-item scales, all items were standardized (in case different response options were used for different items within the scale) and combined into one scale. In case of two-item scales, internal consistency indicator represents Pearson correlation between the items, in case of multi-item scales, internal consistency indicator represents Cronbach's α. Internal consistency indicators are computed using the entire sample.

Supplementary Figure 1

Study 1, Exploratory Sample: Association between attitude at wave 1 and attitude fluctuation over 3 waves (measured as within-person MSSD; the higher the value, the more fluctuation there was)

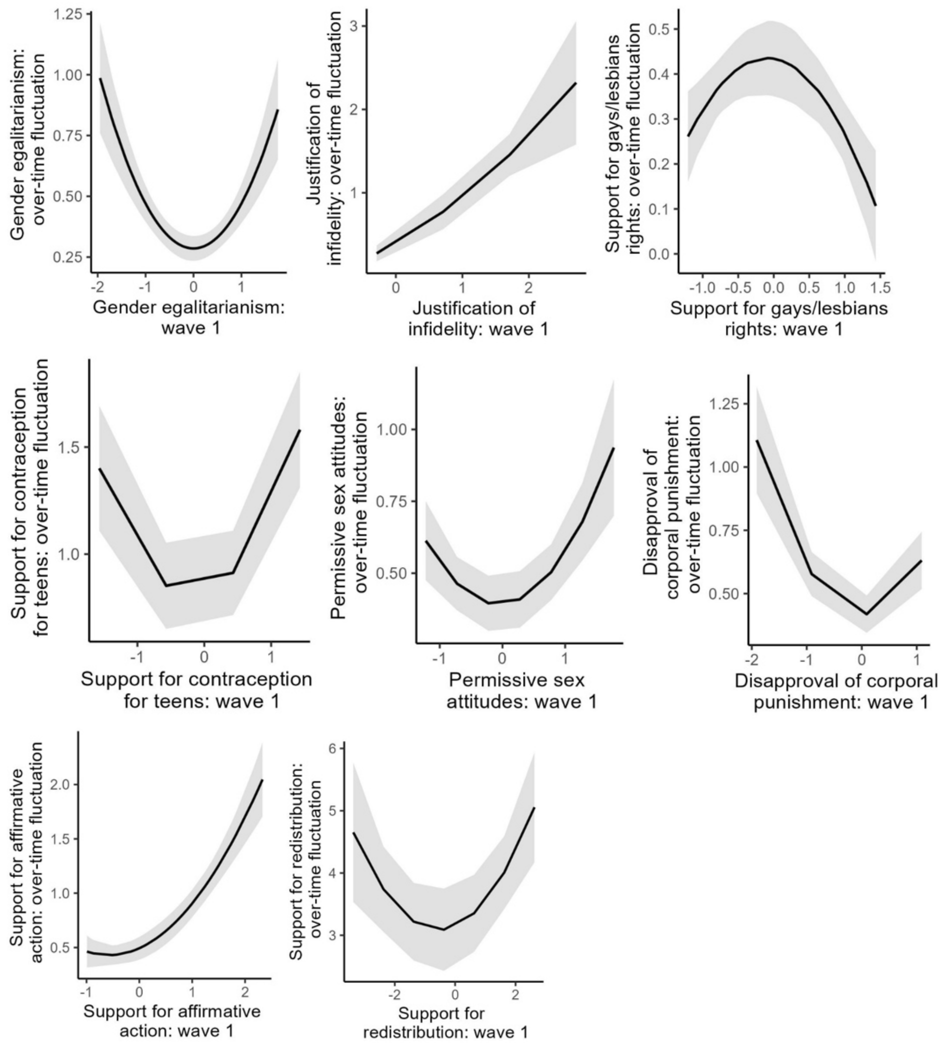

Note. Attitudes at wave 1 were centered around mean; over-time fluctuation is represented by MSSD (higher values reflect more fluctuations, 0 reflects complete stability).

Supplementary Figure 2

Study 1, Exploratory Sample: Attitude change trajectories as a function of initial attitude

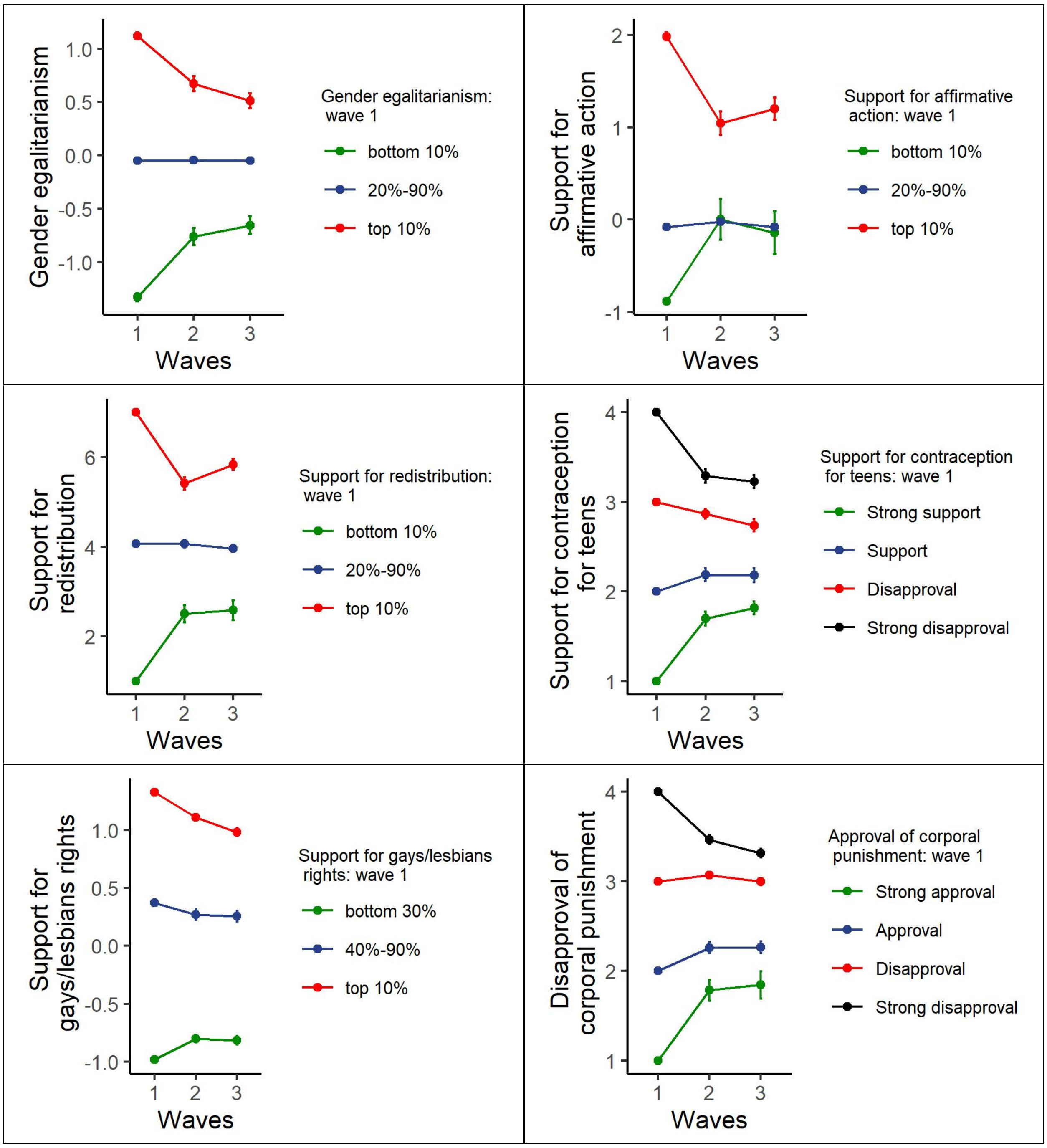

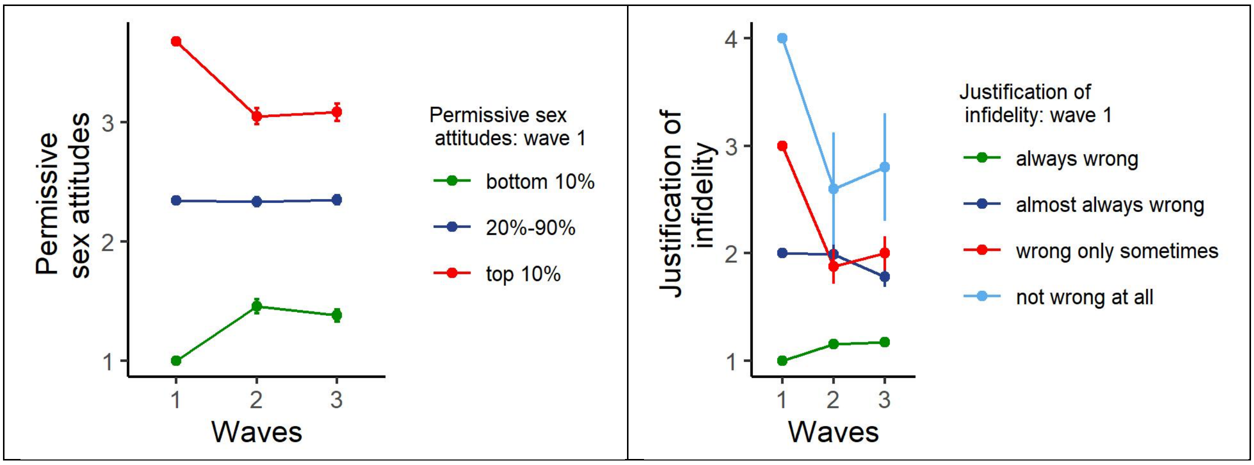

Note. For single-item scales that used 4-point response we present the over-time trajectories for each response category in wave 1, rather than top and bottom 10%. Support for gays/lesbians: there were only 10 participants in the bottom 10% of the distribution (due to skewness), therefore we plotted bottom 30%. Error bars are standard errors (barely visible on most graphs).

Supplementary Figure 3

Study 1: Relationship between attitude similarity and attitude change.

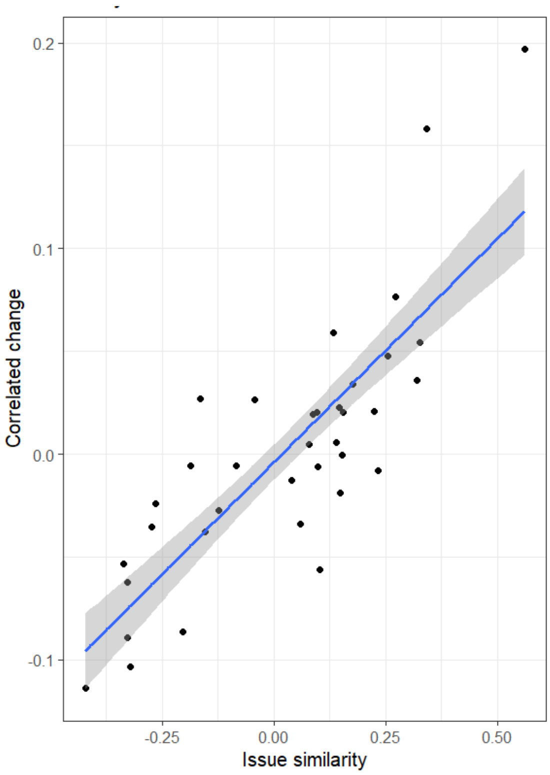

Note. Each point represents a pair of issues (e.g., permissive sex attitudes and support of gays/lesbians); the x-axis shows how similar issues in each pair are at baseline (i.e., do people who scored high on one issue scored high on the other one?) and the y-axis shows how similarly issues in each pair changed over time (i.e., do people who increased on one issue increased on the other one as well?).

Supplementary Table 2

Study 1: Results of quadratic regression models with attitude at wave 1 (linear and quadratic terms) predicting attitude fluctuation over time (measured using *SD*)

		Exploratory sample			Holdout sample		
Attitude		*b*	95% CI	*p*	*b*	95% CI	*p*
Gender egalitarianism	Linear	-0.01	-0.03 – 0.02	0.500	-0.00	-0.01 – 0.01	0.947
	Quadratic	0.10	0.07 – 0.13	**<0.001**	0.07	0.06 – 0.09	**<0.001**
Support for affirmative action	Linear	0.13	0.09 – 0.17	**<0.001**	0.14	0.12 – 0.16	**<0.001**
	Quadratic	0.01	-0.02 – 0.04	0.564	-0.01	-0.03 – 0.00	0.160
Support for redistribution	Linear	0.01	-0.03 – 0.04	0.637	0.03	0.01 – 0.04	**0.004**
	Quadratic	0.01	-0.01 – 0.03	0.315	0.01	-0.00 – 0.02	0.058
Support for contraception for teens	Linear	0.01	-0.03 – 0.04	0.691	0.02	-0.00 – 0.04	0.057
	Quadratic	0.06	0.02 – 0.10	**0.004**	0.06	0.04 – 0.08	**<0.001**
Support of gays/lesbians	Linear	0.01	-0.01 – 0.03	0.403	0.01	-0.00 – 0.02	0.090
	Quadratic	-0.14	-0.18 – -0.11	**<0.001**	-0.14	-0.16 – -0.12	**<0.001**
Disapproval of children corporal punishment	Linear	0.02	-0.01 – 0.06	0.233	-0.01	-0.03 – 0.01	0.437
	Quadratic	0.10	0.07 – 0.13	**<0.001**	0.07	0.05 – 0.09	**<0.001**
Permissive sex attitudes	Linear	0.05	0.01 – 0.08	**0.004**	0.05	0.03 – 0.06	**<0.001**
	Quadratic	0.04	0.01 – 0.07	**0.013**	0.01	-0.00 – 0.03	0.107
Justification of sexual infidelity	Linear	0.38	0.28 – 0.48	**<0.001**	0.29	0.24 – 0.34	**<0.001**
	Quadratic	-0.05	-0.11 – 0.02	0.142	0.02	-0.00 – 0.05	0.099

Note. Attitude fluctuation is measures as standard deviation computed for each participant over the three waves.

Supplementary Table 3

Study 1, whole sample: Tobit model of quadratic regression models with attitude at wave 1 (linear and quadratic terms) predicting attitude change over time (measured using MSSD)

Attitude		*b*	95% CI	*p*
Gender egalitarianism	Linear	-0.01	-0.04 – 0.01	0.355
	Quadratic	0.15	0.13 – 0.18	**<0.001**
Support for affirmative action	Linear	0.17	0.12 – 0.23	**<0.001**
	Quadratic	0.11	0.06 – 0.15	**<0.001**
Support for redistribution	Linear	0.17	0.05 – 0.30	**0.007**
	Quadratic	0.09	0.03 – 0.16	**0.005**
Support for contraception for teens	Linear	0.09	0.02 – 0.17	**0.016**
	Quadratic	0.28	0.20 – 0.36	**<0.001**
Support of gays/lesbians	Linear	-0.01	-0.03 – 0.02	0.642
	Quadratic	-0.16	-0.20 – -0.12	**<0.001**
Disapproval of children corporal punishment	Linear	0.01	-0.06 – 0.07	0.872
	Quadratic	0.26	0.20 – 0.31	**<0.001**
Permissive sex attitudes	Linear	0.16	0.11 – 0.21	**<0.001**
	Quadratic	0.06	0.00 – 0.11	**0.032**
Justification of sexual infidelity	Linear	2.36	1.99 – 2.73	**<0.001**
	Quadratic	-0.33	-0.53 – -0.14	**0.001**
Political ideology	Linear	-0.02	-0.11 – 0.06	0.605
	Quadratic	0.28	0.23 – 0.33	**<0.001**

Supplementary Table 4

Study 1, whole sample: Tobit mode, quadratic moderation analyses

Attitude		*b*	95% CI	*p*
Gender egalitarianism	Time linear	0.10	0.07 – 0.13	**<0.001**
	Time quadratic	-0.12	-0.15 – -0.09	**<0.001**
	Baseline attitude	0.53	0.50 – 0.56	**<0.001**
	Time linear x baseline attitude	-0.35	-0.40 – -0.30	**<0.001**
	Time quadratic x baseline attitude	0.31	0.25 – 0.36	**<0.001**
Support for affirmative action	Time linear	0.14	0.10 – 0.18	**<0.001**
	Time quadratic	-0.14	-0.19 – -0.09	**<0.001**
	Baseline attitude	0.62	0.59 – 0.65	**<0.001**
	Time linear x baseline attitude	-0.33	-0.37 – -0.28	**<0.001**
	Time quadratic x baseline attitude	0.31	0.26 – 0.37	**<0.001**
Support for redistribution	Time linear	-0.16	-0.24 – -0.08	**<0.001**
	Time quadratic	0.05	-0.05 – 0.15	0.306
	Baseline attitude	0.71	0.68 – 0.74	**<0.001**
	Time linear x baseline attitude	-0.44	-0.48 – -0.40	**<0.001**
	Time quadratic x baseline attitude	0.50	0.45 – 0.55	**<0.001**
Support for contraception for teens	Time linear	-0.05	-0.10 – 0.00	0.074
	Time quadratic	0.03	-0.04 – 0.09	0.403
	Baseline attitude	0.87	0.83 – 0.90	**<0.001**
	Time linear x baseline attitude	-0.64	-0.72 – -0.56	**<0.001**
	Time quadratic x baseline attitude	0.48	0.39 – 0.57	**<0.001**
Support of gays/lesbians	Time linear	0.18	0.15 – 0.21	**<0.001**
	Time quadratic	-0.16	-0.19 – -0.12	**<0.001**
	Baseline attitude	0.67	0.64 – 0.71	**<0.001**
	Time linear x baseline attitude	-0.28	-0.33 – -0.23	**<0.001**
	Time quadratic x baseline attitude	0.23	0.17 – 0.28	**<0.001**
Disapproval of children corporal punishment	Time linear	-0.08	-0.11 – -0.04	**<0.001**
	Time quadratic	0.02	-0.02 – 0.06	0.303
	Baseline attitude	0.72	0.70 – 0.75	**<0.001**
	Time linear x baseline attitude	-0.42	-0.47 – -0.38	**<0.001**
	Time quadratic x baseline attitude	0.39	0.34 – 0.45	**<0.001**
Permissive sex attitudes	Time linear	0.08	0.05 – 0.11	**<0.001**
	Time quadratic	-0.05	-0.09 – -0.01	**0.006**
	Baseline attitude	0.82	0.80 – 0.85	**<0.001**
	Time linear x baseline attitude	-0.33	-0.36 – -0.29	**<0.001**
	Time quadratic x baseline attitude	0.31	0.27 – 0.35	**<0.001**
Justification of sexual infidelity	Time linear	0.59	0.38 – 0.80	**<0.001**
	Time quadratic	-0.53	-0.76 – -0.31	**<0.001**
	Baseline attitude	1.25	1.13 – 1.36	**<0.001**
	Time linear x baseline attitude	-0.99	-1.17 – -0.81	**<0.001**
	Time quadratic x baseline attitude	0.95	0.75 – 1.14	**<0.001**
Political ideology	Time linear	0.01	-0.05 – 0.07	0.726
	Time quadratic	0.01	-0.05 – 0.07	0.754
	Baseline attitude	0.65	0.64 – 0.67	**<0.001**
	Time linear x baseline attitude	-0.24	-0.26 – -0.21	**<0.001**
	Time quadratic x baseline attitude	0.21	0.18 – 0.23	**<0.001**

Supplementary Table 5

Attitude items, Study 2, the Longitudinal Internet Studies for the Social Sciences (LISS)

Attitude	Items	Response options	Internal consistency range
Justification of euthanasia	Some people believe that euthanasia should always be forbidden. Others feel that euthanasia should be permitted if the patient expresses that wish. Still others hold an opinion that lies somewhere in between. Where would you place yourself on a scale from 1 to 5, where 1 means that euthanasia should be forbidden and 5 means that euthanasia should be permitted?	1-5; don't know (coded as missing)	-
Support for income equality	Some people believe that differences in income should increase in our country. Others feel that they should decrease. Still others hold an opinion that lies somewhere in between. Where would you place yourself on a scale from 1 to 5, where 1 means that differences in income should increase and 5 means that these should decrease?	1-5; don't know (coded as missing)	-
Support for acculturation	In the Netherlands, some people believe that immigrants are entitled to live here while retaining their own culture. Others feel that they should adapt entirely to Dutch culture. Where would you place yourself on a scale of 1 to 5, where 1 means that immigrants can retain their own culture and 5 means that they should adapt entirely?	1-5; don't know (coded as missing)	-
Disapproval of EU unification	Some people and political parties feel that European unification should go a step further. Others think that European unification has already gone too far. Where would you place yourself on a scale from 1 to 5, where 1 means that European unification should go further and 5 means that it has already gone too far?	1-5; don't know (coded as missing)	-

Gender egalitarianism in family	• A working mother's relationship with her children can be just as close and warm as that of a non-working mother.<br>• A child that is not yet attending school is likely to suffer the consequences if his or her mother has a job. *(reverse-coded)*<br>• Overall, family life suffers the consequences if the mother has a full-time job. *(reverse-coded)*<br>• Both father and mother should contribute to the family income.<br>• The father should earn money, while the mother takes care of the household and the family. *(reverse-coded)*<br>• Fathers ought to do more in terms of household work than they do at present.<br>• Fathers ought to do more in terms of childcare than they do at present.	1. fully disagree<br>2. disagree<br>3. neither agree nor disagree<br>4. agree<br>5. fully agree	α: .75-.78
Gender egalitarianism at work	• A woman is more suited to rearing young children than a man. *(reverse-coded)*<br>• It is actually less important for a girl than for a boy to get a good education. *(reverse-coded)*<br>• Generally speaking, boys can be reared more liberally than girls. *(reverse-coded)*<br>• It is unnatural for women in firms to have control over men. *(reverse-coded)*	1. fully disagree<br>2. disagree<br>3. neither agree nor disagree<br>4. agree<br>5. fully agree	α: .69-.72
Multiculturalism	• It is good if society consists of people from different cultures.<br>• It should be made easier to obtain asylum in the Netherlands.<br>• Legally residing foreigners should be entitled to the same social security as Dutch citizens.<br>• There are too many people of foreign origin or descent in the Netherlands. *(reverse-coded)*<br>• Some sectors of the economy can only continue to function because people of foreign origin or descent work there.	1. fully disagree<br>2. disagree<br>3. neither agree nor disagree<br>4. agree<br>5. fully agree	α: .77-.81

	• It does not help a neighborhood if many people of foreign origin or descent move in. *(reverse-coded)*		
Approval of inter-generational support	• Children ought to care for their sick parents.<br>• When parents reach old age, they should be able to live with their children.<br>• Children that live close by ought to visit their parents at least once a week.<br>• Children ought to take unpaid leave in order to care for their sick parents.	1. fully disagree<br>2. disagree<br>3. neither agree nor disagree<br>4. agree<br>5. fully agree	α: .69-.76
Approval of marriage	• Married people are generally happier than unmarried people.<br>• People that want to have children should get married.<br>• A single parent can raise a child just as well as two parents together. *(reverse-coded)*<br>• It is perfectly fine for a couple to live together without marriage intentions. *(reverse-coded)*	1. fully disagree<br>2. disagree<br>3. neither agree nor disagree<br>4. agree<br>5. fully agree	α: .67-.69
Justification of divorce	• A divorce is generally the best solution if a married couple cannot solve their marital problems.<br>• It is all right for a married couple with children to get divorced	1. fully disagree<br>2. disagree<br>3. neither agree nor disagree<br>4. agree<br>5. fully agree	*r*: .43-.49

Note. Items were reverse-coded when necessary. For multiple-item scales, all items were combined into one scale. In case of two-item scales, internal consistency indicator represents Pearson correlation between the items, in case of multi-item scale, internal consistency indicators represent Cronbach's α. Internal consistency indicators are computed using the entire sample.

Supplementary Figure 4

Study 2, Exploratory Sample: Association between attitude at wave 1 and attitude change over 13 waves (measured as within-person SD)

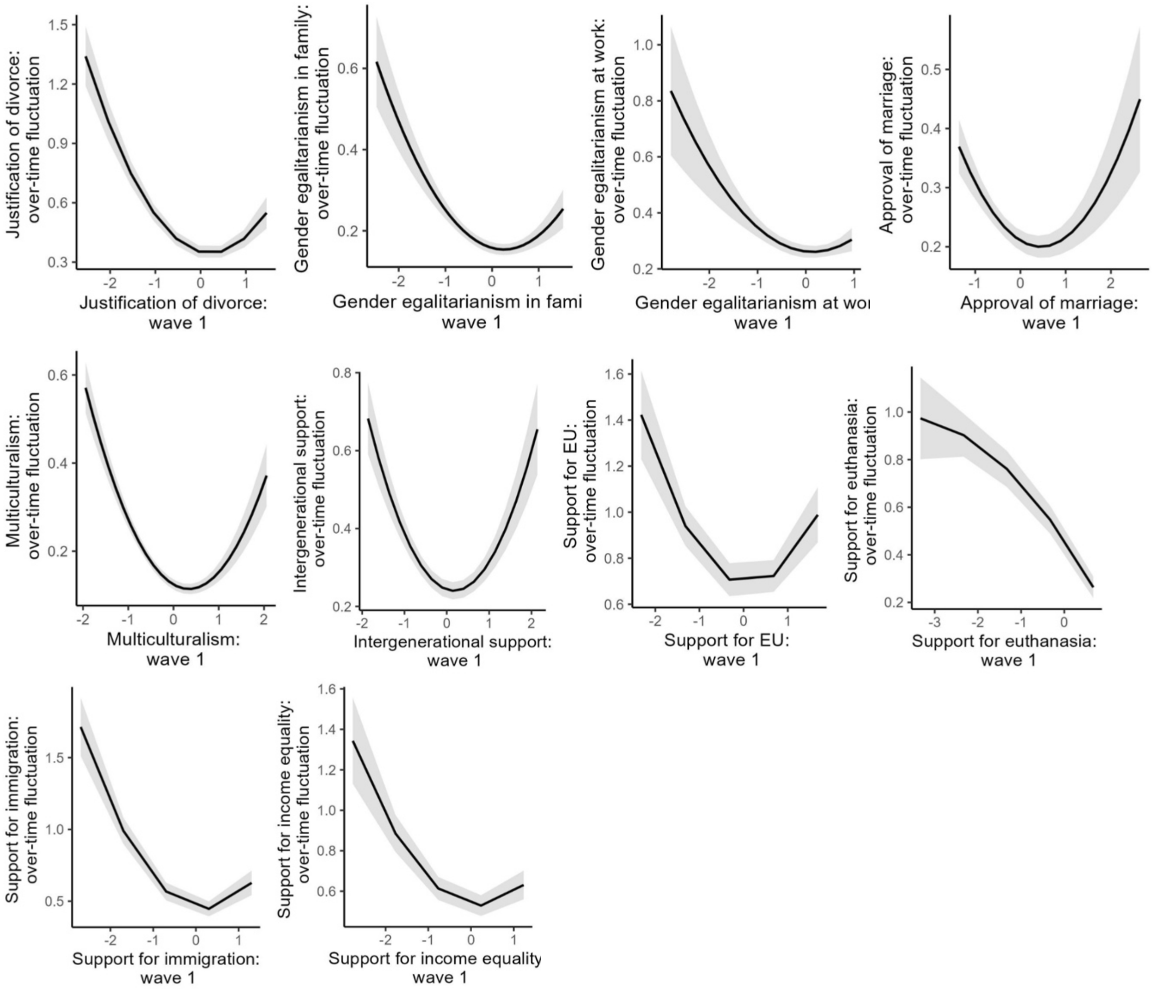

Note. Attitudes at wave 1 were centered around mean; over-time fluctuation is represented MSSD (higher values reflect more fluctuations, 0 reflects complete stability).

Supplementary Table 6

Study 2: Results of quadratic regression models with attitude at wave 1 (linear and quadratic terms) predicting attitude fluctuations over time (measured using *SD*).

		Exploratory sample			Holdout sample		
Attitude		*b*	95% CI	*p*	*b*	95% CI	*p*
Justification of euthanasia	Linear	-0.23	-0.26 – -0.21	**<0.001**	-0.27	-0.28 – -0.25	**<0.001**
	Quadratic	-0.05	-0.07 – -0.04	**<0.001**	-0.08	-0.08 – -0.07	**<0.001**
Support for income equality	Linear	-0.03	-0.05 – -0.02	**<0.001**	-0.03	-0.04 – -0.03	**<0.001**
	Quadratic	0.04	0.02 – 0.05	**<0.001**	0.05	0.04 – 0.05	**<0.001**
Support for acculturation	Linear	-0.04	-0.05 – -0.02	**<0.001**	-0.03	-0.04 – -0.02	**<0.001**
	Quadratic	0.05	0.03 – 0.06	**<0.001**	0.04	0.03 – 0.05	**<0.001**
Disapproval of EU unification	Linear	-0.02	-0.04 – -0.01	**0.006**	-0.03	-0.04 – -0.02	**<0.001**
	Quadratic	0.03	0.02 – 0.04	**<0.001**	0.03	0.02 – 0.03	**<0.001**
Gender egalitarianism in family	Linear	-0.03	-0.04 – -0.02	**<0.001**	-0.02	-0.02 – -0.01	**<0.001**
	Quadratic	0.04	0.03 – 0.05	**<0.001**	0.04	0.03 – 0.04	**<0.001**
Gender egalitarianism at work	Linear	-0.04	-0.06 – -0.03	**<0.001**	-0.05	-0.05 – -0.04	**<0.001**
	Quadratic	0.04	0.02 – 0.06	**<0.001**	0.04	0.04 – 0.05	**<0.001**
Multiculturalism	Linear	-0.05	-0.06 – -0.04	**<0.001**	-0.04	-0.04 – -0.03	**<0.001**
	Quadratic	0.06	0.05 – 0.07	**<0.001**	0.05	0.04 – 0.05	**<0.001**
Approval of intergenerational support	Linear	-0.01	-0.03 – -0.00	**0.022**	-0.00	-0.01 – 0.00	0.135
	Quadratic	0.07	0.06 – 0.08	**<0.001**	0.07	0.07 – 0.08	**<0.001**
Approval of marriage	Linear	-0.01	-0.02 – -0.00	**0.030**	-0.01	-0.01 – -0.00	**0.002**
	Quadratic	0.03	0.02 – 0.04	**<0.001**	0.03	0.02 – 0.04	**<0.001**
Justification of divorce	Linear	-0.04	-0.05 – -0.03	**<0.001**	-0.03	-0.04 – -0.02	**<0.001**
	Quadratic	0.05	0.04 – 0.06	**<0.001**	0.06	0.05 – 0.07	**<0.001**

Note. Attitude fluctuation is measures as standard deviation computed for each participant over the three waves.

Supplementary Figure 5

Study 2, Exploratory Sample: Attitude change trajectories as a function of initial attitude.

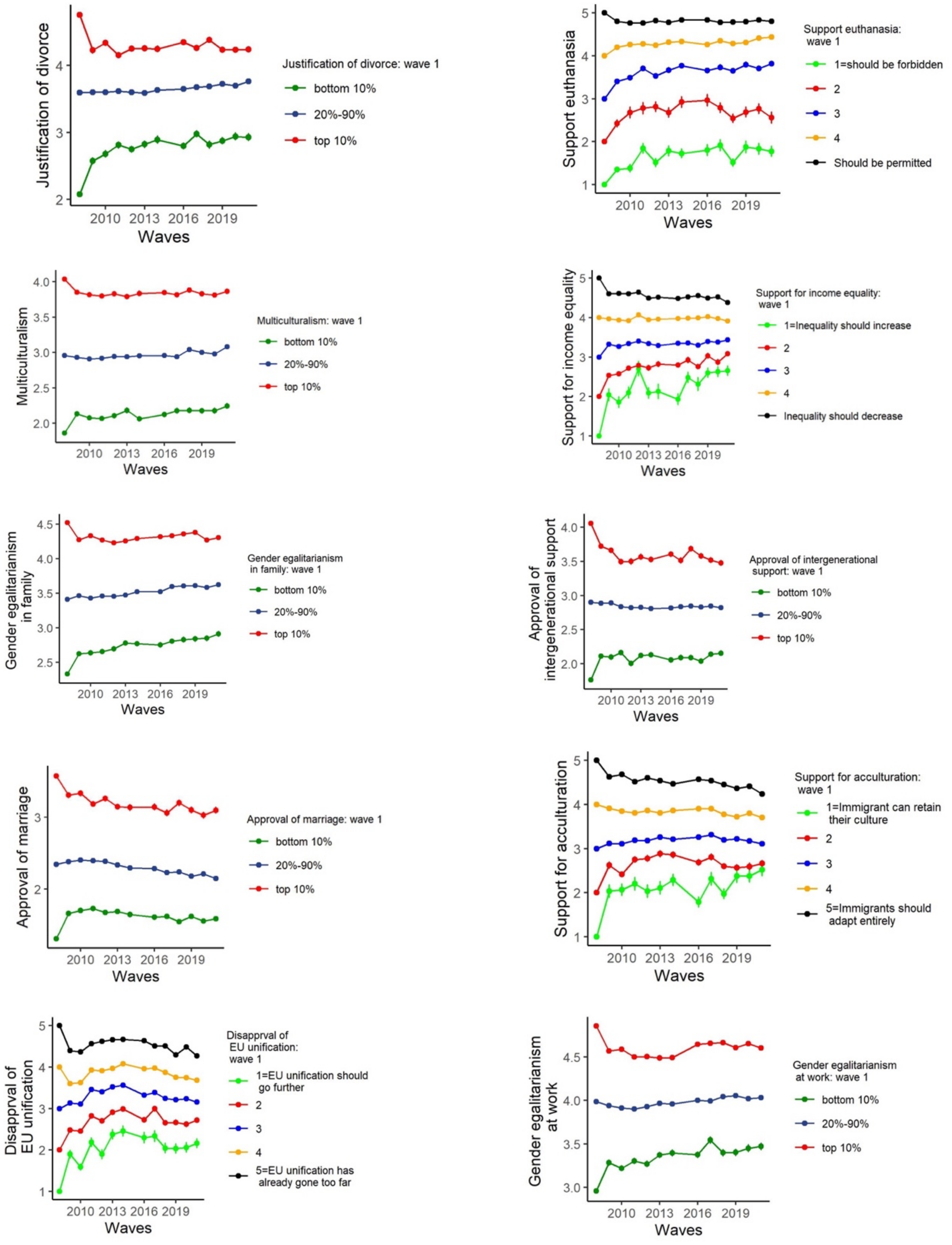

Note. Attitudes measured with single items: developmental trajectory associated with each response category is shown; attitudes measured with multi-item scales: developmental trajectories of bottom 10%, middle 20%-90% and top 10% are shown. Each point includes error bars that represent standard errors (too small to be discerned).

Supplementary Figure 6

Study 2: Relationship between attitude similarity and attitude change.

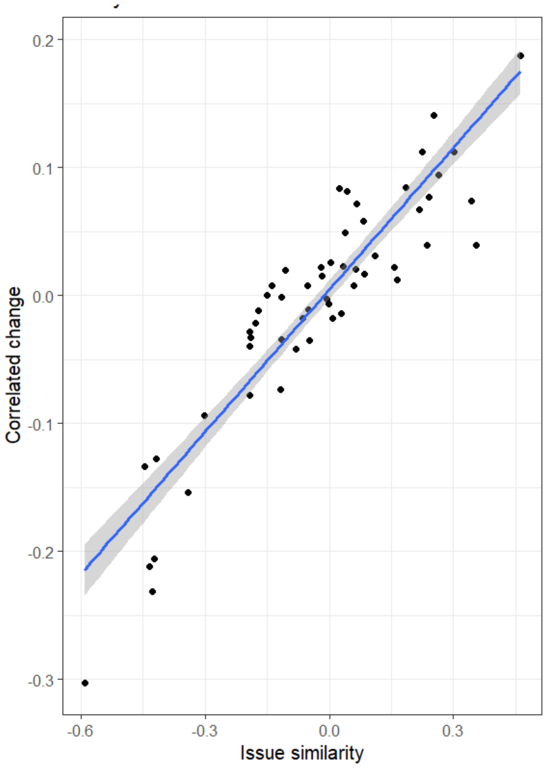

Note. Each point represents a pair of issues (e.g., permissive sex attitudes and support of gays/lesbians); the x-axis shows how similar issues in each pair are at baseline (i.e., do people who scored high on one issue scored high on the other one?) and the y-axis shows how similarly issues in each pair changed over time (i.e., do people who increased on one issue increased on the other one as well?).

Supplementary Table 7

Study 2, whole sample: Tobit model, results of quadratic regression models with attitude at wave 1 (linear and quadratic terms) predicting attitude change over time (measuring with MSSD)

Attitude		*b*	95% CI	*p*
Justification of euthanasia	Linear	-0.83	-0.88 – -0.79	**<0.001**
	Quadratic	-0.23	-0.25 – -0.21	**<0.001**
Support for income equality	Linear	-0.09	-0.11 – -0.06	**<0.001**
	Quadratic	0.14	0.12 – 0.16	**<0.001**
Support for acculturation	Linear	-0.11	-0.13 – -0.08	**<0.001**
	Quadratic	0.14	0.12 – 0.16	**<0.001**
Disapproval of EU unification	Linear	-0.06	-0.09 – -0.04	**<0.001**
	Quadratic	0.11	0.08 – 0.13	**<0.001**
Gender egalitarianism in family	Linear	-0.01	-0.02 – -0.01	**<0.001**
	Quadratic	0.05	0.04 – 0.06	**<0.001**
Gender egalitarianism at work	Linear	-0.05	-0.07 – -0.04	**<0.001**
	Quadratic	0.09	0.07 – 0.10	**<0.001**
Multiculturalism	Linear	-0.04	-0.05 – -0.04	**<0.001**
	Quadratic	0.07	0.06 – 0.07	**<0.001**
Approval of intergenerational support	Linear	-0.02	-0.03 – -0.01	**0.004**
	Quadratic	0.13	0.12 – 0.14	**<0.001**
Approval of marriage	Linear	-0.02	-0.03 – -0.01	**<0.001**
	Quadratic	0.04	0.03 – 0.05	**<0.001**
Justification of divorce	Linear	-0.05	-0.07 – -0.03	**<0.001**
	Quadratic	0.13	0.11 – 0.14	**<0.001**
Political ideology	Linear	-0.04	-0.09 – 0.01	0.109
	Quadratic	0.14	0.13 – 0.16	**<0.001**

Supplementary Table 8

Study 2, whole sample: Tobit model, quadratic moderation analyses

Attitude		*b*	95% CI	*p*
Justification of euthanasia	Time linear	0.02	0.02 – 0.02	**<0.001**
	Time quadratic	0.00	-0.00 – 0.00	0.091
	Baseline attitude	1.32	1.30 – 1.34	**<0.001**
	Time linear x baseline attitude	-0.04	-0.04 – -0.04	**<0.001**
	Time quadratic x baseline attitude	0.01	0.01 – 0.01	**<0.001**
Support for income equality	Time linear	0.01	0.00 – 0.01	**<0.001**
	Time quadratic	0.00	0.00 – 0.00	**0.004**
	Baseline attitude	0.78	0.76 – 0.79	**<0.001**
	Time linear x baseline attitude	-0.04	-0.04 – -0.04	**<0.001**
	Time quadratic x baseline attitude	0.00	0.00 – 0.01	**<0.001**
Support for acculturation	Time linear	-0.01	-0.02 – -0.01	**<0.001**
	Time quadratic	-0.00	-0.00 – -0.00	**<0.001**
	Baseline attitude	0.76	0.75 – 0.77	**<0.001**
	Time linear x baseline attitude	-0.04	-0.04 – -0.04	**<0.001**
	Time quadratic x baseline attitude	0.00	0.00 – 0.00	**<0.001**
Disapproval of EU unification	Time linear	0.01	0.01 – 0.01	**<0.001**
	Time quadratic	-0.01	-0.01 – -0.01	**<0.001**
	Baseline attitude	0.83	0.82 – 0.84	**<0.001**
	Time linear x baseline attitude	-0.03	-0.03 – -0.03	**<0.001**
	Time quadratic x baseline attitude	0.00	0.00 – 0.00	**<0.001**
Gender egalitarianism in family	Time linear	0.01	0.01 – 0.01	**<0.001**
	Time quadratic	0.00	-0.00 – 0.00	0.812
	Baseline attitude	0.69	0.68 – 0.70	**<0.001**
	Time linear x baseline attitude	-0.02	-0.02 – -0.02	**<0.001**
	Time quadratic x baseline attitude	0.00	0.00 – 0.00	**<0.001**
Gender egalitarianism at work	Time linear	0.01	0.01 – 0.01	**<0.001**
	Time quadratic	0.00	0.00 – 0.00	**<0.001**
	Baseline attitude	0.68	0.67 – 0.69	**<0.001**
	Time linear x baseline attitude	-0.02	-0.02 – -0.02	**<0.001**
	Time quadratic x baseline attitude	0.00	0.00 – 0.00	**<0.001**
Multiculturalism	Time linear	0.00	0.00 – 0.00	**<0.001**
	Time quadratic	0.00	0.00 – 0.00	**<0.001**
	Baseline attitude	0.78	0.77 – 0.78	**<0.001**
	Time linear x baseline attitude	-0.01	-0.02 – -0.01	**<0.001**
	Time quadratic x baseline attitude	0.00	0.00 – 0.00	**<0.001**
Approval of intergenerational support	Time linear	-0.01	-0.01 – -0.01	**<0.001**
	Time quadratic	0.00	0.00 – 0.00	**<0.001**
	Baseline attitude	0.62	0.62 – 0.63	**<0.001**
	Time linear x baseline attitude	-0.02	-0.02 – -0.02	**<0.001**
	Time quadratic x baseline attitude	0.00	0.00 – 0.00	**<0.001**
Approval of marriage	Time linear	-0.02	-0.02 – -0.02	**<0.001**
	Time quadratic	-0.00	-0.00 – -0.00	**<0.001**
	Baseline attitude	0.71	0.70 – 0.71	**<0.001**
	Time linear x baseline attitude	-0.02	-0.02 – -0.02	**<0.001**
	Time quadratic x baseline attitude	0.00	0.00 – 0.00	**<0.001**
Justification of divorce	Time linear	0.01	0.01 – 0.01	**<0.001**
	Time quadratic	0.00	0.00 – 0.00	**<0.001**
	Baseline attitude	0.61	0.61 – 0.62	**<0.001**
	Time linear x baseline attitude	-0.03	-0.03 – -0.03	**<0.001**
	Time quadratic x baseline attitude	0.00	0.00 – 0.00	**<0.001**
Political ideology	Time linear	0.00	-0.00 – 0.00	0.996
	Time quadratic	-0.00	-0.00 – -0.00	**0.003**
	Baseline attitude	0.76	0.75 – 0.77	**<0.001**
	Time linear x baseline attitude	-0.02	-0.02 – -0.02	**<0.001**
	Time quadratic x baseline attitude	0.00	0.00 – 0.00	**<0.001**

Supplementary Table 9

Study 3: Perceptions of attitude change among extremists and moderates.

Issue	Target person's attitude	Target person's stance	Perceptions of future attitude change
Affirmative Action	Extreme	Oppose	1.99_a (1.28)
	Moderate	Oppose	3.67_b (1.43)
	Extreme	Support	1.79_a (.88)
	Moderate	Support	3.43_b (1.40)
Wealth redistribution	Extreme	Oppose	2.16_a (1.32)
	Moderate	Oppose	3.59_b (1.28)
	Extreme	Support	1.86_a (1.00)
	Moderate	Support	3.58_b (1.16)
Gay marriage	Extreme	Oppose	1.69_a (.93)
	Moderate	Oppose	2.19_b (1.20)
	Extreme	Support	1.44_a (.92)
	Moderate	Support	2.21_b (1.26)

Note. Standard deviations are in parentheses. Subscripts within each policy issue denote means that differ at $p < .05$.
